# COVID-19 Vaccination: Comparison of Attitudes, Decision-Making Processes, and Communication among Vaccinated and Unvaccinated Black Americans

**DOI:** 10.3390/ijerph20043481

**Published:** 2023-02-16

**Authors:** Jennifer Cunningham-Erves, Whitney George, Elizabeth C. Stewart, Alison Footman, Jamaine Davis, Maureen Sanderson, Meredith Smalls, Phillip Morris, Kristin Clarkson, Omaran Lee, Heather M. Brandt

**Affiliations:** 1 Department of Internal Medicine, Meharry Medical College, School of Medicine, 1005 Dr. D.B. Todd Jr. Blvd, Nashville, TN 37208, USA; 2 Vanderbilt University Medical Center, 1211 Medical Center Drive, Nashville, TN 27232, USA; 3 Department of Epidemiology and Cancer Control, St. Jude Children’s Research Hospital, 262 Danny Thomas Place, Memphis, TN 38105, USA; 4 Department of Biochemistry and Cancer Biology, School of Medicine, Meharry Medical College, 1005 Dr. D.B. Todd Jr. Blvd, Nashville, TN 37208, USA; 5 Department of Family and Community Medicine, School of Medicine, Meharry Medical College, 1005 Dr. D.B. Todd Jr. Blvd, Nashville, TN 37208, USA; 6 Meharry Vanderbilt Alliance, 1903 Meharry Boulevard, Nashville, TN 37208, USA; 7 Congregational Health & Education Network, 1818 Albion St, Nashville, TN 37208, USA; 8 Centers for Wellbeing, P.O. Box 330191, Nashville, TN 37203, USA

**Keywords:** COVID-19, vaccine hesitancy, Black Americans, communication, culturally appropriate

## Abstract

Background: COVID-19 vaccination rates remain suboptimal among Black Americans who disproportionately experience higher hospitalization and death rates than White Americans. Methods: We conducted a multi-method (interviews and surveys) study among 30 Black Americans (*n* = 16 vaccinated, *n* = 14 unvaccinated) to explore factors related to vaccination hesitancy, decision-making processes, and communication related to uptake. Participants were recruited by using community-driven approaches, including partner collaborations. Thematic analysis was used to analyze qualitative data, and descriptive and bivariate analysis was used for quantitative data. Results: Of those unvaccinated, 79% (*n* = 11) stated they were delaying and 21% (*n* = 3) were declining vaccination indefinitely. When asked about the likelihood of vaccine initiation in 6 months and 12 months, 29% (*n* = 4) and 36% (*n* = 5), respectively, stated that they would receive the vaccine. The following themes emerged: (1) COVID-19 vaccination hesitancy exists on a continuum; (2) varied decision-making processes for COVID-19 vaccination; (3) motivators among vaccinated individuals; (4) barriers among unvaccinated individuals; (5) retrieving and navigating vaccine information within the COVID-19 infodemic; and (6) parent perspectives on child vaccination. Conclusions: Findings suggest that vaccinated and unvaccinated participants had similar and dissimilar perspectives in decision-making processes and vaccine concerns as shown in the Decision-making Processes for the COVID-19 vaccination (DePC) model. Based on these findings, future studies should further explore how factors influencing decision-making can lead to divergent outcomes for COVID-19 vaccination.

## 1. Introduction

Across the United States (US), COVID-19-related health disparities exist, with Black Americans more likely than any other racial or ethnic groups to experience hospitalization and death [1]. Black Americans represent 12.5% of the US population and 13.1% of COVID-19-related deaths reported as of 3 February, 2023 [2]. Black Americans are 1.7 times and 1.8 times more likely to die and be hospitalized from COVID-19 compared to White Americans. Multiple vaccines have been approved for use since December 2020. Vaccination significantly reduces the risk of severe illness, hospitalization, and death due to COVID-19 [3,4]. Despite the availability of these vaccines and the protection they offer, uptake remains suboptimal among Black Americans. Only 51.1% initiated the primary series, and 44.8% completed the primary series from 14 December 2020 to 1 February 2023 [5]. Increasing vaccine uptake among Black Americans is crucial to address the disproportionate impact of COVID-19 among this population [6,7,8].

Vaccine hesitancy is a major barrier to COVID-19 vaccine uptake [9]. Multiple factors influence uptake, and vaccine hesitancy varies by socio-demographic factors, including age, sex, and race. Studies continue to demonstrate that COVID-19 vaccine hesitancy is disproportionately higher among Black Americans than among other racial and ethnic groups [10,11]. These studies confirm that vaccine hesitancy remains a public health threat [12]. Needs and strategies must be explored by using community engagement, a strategy that creates positive behavior change across many behaviors [13,14,15]. Furthermore, no studies to date have qualitatively explored factors influencing vaccine uptake and communication about the vaccine from both vaccinated and unvaccinated individuals, which is critical for strategy and intervention development.

Many studies have identified the need for educational, communication, and behavioral interventions to address COVID-19 vaccine hesitancy among Black Americans [16,17,18,19]. These interventions should be culturally appropriate [15,17] to maximize ‘fit’. Culturally targeted interventions have been successful in improving health behaviors among Black Americans [6,20,21]. However, there is a lack of research on processes for decision-making for COVID-19 vaccination to enhance cultural relevance of interventions to motivate uptake.

The purpose of this multi-method study was to explore and compare factors related to COVID-19 vaccine hesitancy among vaccinated and unvaccinated Black American adults. We also explored decision-making processes and communications related to COVID-19 vaccination. The larger goal of this study is to inform theory-based, culturally appropriate strategies and messaging for a social marketing campaign. Additionally, community-engaged research principles were applied to develop and implement this study to ensure cultural sensitivity.

## 2. Materials and Methods

### 2.1. Project Partners

Meharry Medical College and St. Jude Children’s Research Hospital, two academic research institutions, formed a partnership with Congregational Health and Education Network (CHEN) [22] in 2021. The purpose of this partnership was to build confidence in COVID-19 vaccines among Black Americans in Nashville/Davidson County, Tennessee. CHEN, a faith based non-profit organization, is housed at Nashville General Hospital at Meharry. Its mission is to reduce health disparities among Nashville’s residents by elevating educational attainment and health literacy through faith-based partnerships.

### 2.2. Community Advisory Board

The community advisory panel was composed of eight residents of Nashville/Davidson County, Tennessee. They represented various institutions, community, and health organizations. Panel members met quarterly to (1) discuss the project’s progress; (2) provide insight on vaccine hesitancy or vaccine confidence in the community; and (3) identify strategies to provide accurate and timely information to the community. Panel membership was instrumental in informing the interview protocol and recruitment plan for study participants.

### 2.3. Research Design

We used multiple methods to assess COVID-19 vaccine hesitancy among Black Americans. This study also explored factors related to the communication strategies needed to address hesitancy in vaccinated and unvaccinated Black Americans. Researchers first collected and analyzed survey data (quantitative), and then conducted semi-structured interviews (qualitative) with survey respondents. This approach was chosen to provide an in-depth understanding of this phenomenon.

### 2.4. Conceptual Framework

The Strategic Advisory Group of Experts (SAGE) on vaccine hesitancy developed the 3Cs model: complacency, confidence, and convenience [23]. The constructs provide a framework for assessing population-specific barriers. Complacency is the belief that risks related to vaccine-preventable diseases are low, making vaccination unnecessary. Confidence is the degree of trust in the safety and effectiveness of the vaccine, the need for vaccination, the healthcare system and its related professionals who deliver the vaccine, and the motivations of those who make the policy-related decisions. Convenience is the influence of factors such as physical and geographical availability, affordability, health literacy, and their effect on vaccine uptake.

The 3Cs model considers the perspectives and factors required to understand and influence vaccine hesitancy. Behavior-change models and theories seek to explain the likelihood or intention of a behavior. The health belief model (HBM) [24] and transtheoretical model (TTM) [25] were adapted for this study’s conceptual framework to explore factors influencing COVID-19 vaccine uptake among Black Americans. HBM identifies five influences on health decision-making: (1) perceived severity of a health outcome, (2) perceived susceptibility of being affected by the outcome, (3) benefits of making a preventive choice, (4) barriers to enacting the chosen behavior, and (5) self-efficacy or confidence in being able to perform the behavior. TTM posits that behavior change occurs through six phases (i.e., precontemplation, contemplation, preparation, action, maintenance, and termination) and ten processes (i.e., consciousness raising, counterconditioning, dramatic relief, environmental reevaluation, helping relationships, reinforcement management, self-liberation, self-reevaluation, social liberation, and stimulus control). For this study, we adapted the theoretical underpinnings of the processes of change and decisional balance to understand COVID-19 vaccination decision-making from adults and parents.

### 2.5. Sampling and Recruitment

We recruited a purposive sample of 30 adults. In total, 16 self-reported receiving at least one dose of COVID-19 vaccine, and 14 reported being unvaccinated. Participants were eligible if they were aged 18 years or older and self-identified as Black. Study participants were recruited through community-driven approaches identified by CHEN, the advisory panel, and ResearchMatch [26]. Community-driven approaches included emails from listservs, phone calls, or word of mouth. Specifically, organizations and individuals with whom CHEN and panel membership had a relationship spread the word on the study. Recruitment also occurred using an existing database of past research participants who indicated that they wanted to be contacted for future research studies.

### 2.6. Quantitative

We administered a cross-sectional survey to 30 Black Americans. The measures included socio-demographic information, vaccine status, degree of hesitancy, along with the motivators and barriers to COVID-19 vaccination depending on participants’ status. Survey data were collected and managed by using REDCap [27,28].

#### 2.6.1. Survey Development

The survey was developed by using validated scales, including the Vaccination Confidence Scale [29] and socio-demographic variables. Measures are described below.

1. Socio-Demographics

Participants self-reported their race/ethnicity, sex, education level, age, and household income. If participants had children aged 5 to 17 years, then they reported the race/ethnicity and sex of the youngest child in each age range.

2. Vaccine Status

Participants were asked to report their COVID-19 vaccine status. If participants self-identified as a parent, then they were asked whether their child aged 5 to 17 years old was vaccinated. Response options were ‘Yes’ or ‘No.’

3. Degree of COVID-19 Vaccine Hesitancy

Individuals who reported that they had not received a COVID-19 vaccine identified whether they were delaying vaccination or declining it indefinitely. Those delaying or declining vaccination were then asked how likely they were to receive the vaccine in the next 6 months and within the next year. Those who identified as a parent were asked the same questions about their child. Answer choices were based on a five-point Likert scale.

4. Factors influencing COVID-19 Vaccine Hesitancy

Unvaccinated participants were asked their reasons for COVID-19 vaccine hesitancy. This scale included 11 items and response options. Items included ‘I wonder if getting many different vaccines could cause harm to my health’ or ‘I wonder if natural immunity is better than getting the COVID-19 vaccine.’ If participants were vaccinated, then they were asked to identify reasons they received the COVID-19 vaccine. A list of 12 reasons were given and participants selected whether the factor influenced their decision. Examples of reasons for vaccination provided included ‘a family member has been diagnosed with COVID-19,’ ‘requirement for work,’ and ‘seeing a public figure get a vaccine.’

#### 2.6.2. Data Collection

All participants completed a screening questionnaire to assess eligibility. If qualified, they completed informed consent and brief 10 min survey on barriers to or facilitators of COVID-19 vaccination for themselves and their child(ren), if any.

#### 2.6.3. Data Analysis

SPSS version 28.0 was used to analyze the survey data. To describe patterns in the data, we used descriptive analysis (e.g., means, frequencies) and bivariate analysis (e.g., Chi-square, Fisher’s exact tests, and Spearman’s rho).

### 2.7. Qualitative

#### 2.7.1. Interview Protocol

We drafted an open-ended interview protocol for adults and parents of children aged 5 to 17 years to elicit (1) attitudes, facilitators, and barriers to COVID-19 vaccination; (2) current and preferred information on COVID-19 and the vaccine; and (3) preferred information sources and channels (e.g., radio/television, websites, texts). Protocol development was guided by the 3Cs model, HBM, TTM, cultural targeting strategies, our community partner, and advisory panel input.

#### 2.7.2. Data Collection

Interviews of study participants were scheduled and conducted by a member of the research team and lasted approximately 45–60 min. Data collection occurred between October 2021 and January 2022. Interviews were recorded, transcribed, and de-identified for data analysis. All participants received an incentive for their time. Audio files were submitted to an IRB-approved transcription service (https://rev.com). Each participant was assigned a unique participant identification number.

#### 2.7.3. Data Analysis

The Vanderbilt University Qualitative Research Core, led by a PhD-level psychologist, managed the qualitative data. COREQ guidelines, an evidence-based qualitative methodology, guided data coding, and analysis [30]. The interview guide and a preliminary review of the transcripts were used to develop and refine a hierarchical coding system. Reliability was established in the coding system by experienced qualitative coders first using the coding system on two transcripts, resolving any discrepancies, and then coding the remaining transcripts independently. Using an iterative inductive/deductive approach, we inductively used the coding category to sort the quotes until coding saturation was met. We then performed comparative analysis of those vaccinated and unvaccinated to identify higher-order themes and relationships between themes. Next, we were deductively guided by the 3Cs model, HMB, and TTM. Microsoft Excel 2016 and SPSS version 28.0 were used to manage the transcripts, quotations, and codes.

## 3. Results

### 3.1. Quantitative

#### 3.1.1. Socio-Demographics

Among the study population, both unvaccinated and vaccinated, the majority were female, had a college degree or higher, and earned less than $80,000 per year. The mean age was 38 years. There was no significant difference in vaccination status by demographic information (Table 1).

Of those participants who were unvaccinated (*n* = 14), 11 (78.6%) stated that they were delaying vaccination, and 3 (21.4%) stated they were declining it indefinitely. When asked about the likelihood that they would have the vaccine in 6 months and in the next year, 29% (*n* = 4) and 36% (*n* = 5), respectively, stated that they would receive it.

#### 3.1.2. Vaccination Concerns

The top three concerns among those unvaccinated were as follows: (1) the vaccine is too new (92%), (2) safety issues (85%), and (3) preference for natural immunity over vaccine-induced immunity (85%). Among those who were vaccinated, their top three concerns were as follows: (1) the vaccine could cause serious health problems (69%), (2) how fast the vaccines were made (69%), and (3) whether the vaccine was needed to prevent COVID-19 (63%). There was a statistically significant difference in the following vaccine concerns by vaccination status: preference for natural immunity over vaccine-induced immunity (*p* < 0.001), too many vaccines can harm ones’ health (*p* = 0.017), and the vaccine is too new (*p* = 0.044); see Table 2.

#### 3.1.3. Motivators to COVID-19 Vaccination

Among those who were vaccinated, the top motivators were reading and listening to a news story discussing COVID-19 vaccine trials (43.8%), a friend or family member being diagnosed with COVID-19 (37.5%), a friend or family member receiving COVID-19 vaccine (31.3%), and having a conversation with friends or family about whether to have the vaccine (31.3%) (Table 3).

#### 3.1.4. Barriers to COVID-19 Vaccination

We further explored issues related to COVID-19 vaccine safety. Top barriers to getting COVID-19 vaccination were concerns about developing heart problems (72.7%), infertility (63.6%), and the speed of vaccine development (45.5%) (Table 4).

### 3.2. Qualitative

Six major themes with related subthemes emerged from the data to demonstrate the COVID-19 vaccine decision-making process among Black Americans. The themes were (1) hesitancy for COVID-19 vaccination occurred on a continuum; (2) varied decision-making processes for COVID-19 vaccination; (3) motivators among vaccinated individuals; (4) barriers among unvaccinated individuals; (5) retrieving and navigating vaccine information within the COVID-19 infodemic; and (6) parent perspectives on vaccinating children. Each theme, related subthemes, and participant quotes are detailed below.

#### 3.2.1. Hesitancy for COVID-19 Vaccination Occurred on a Continuum

In exploring the vaccine hesitancy continuum among Black American participants, participants’ vaccination statuses represented the full continuum. Some participants accepted the COVID-19 vaccine with no doubt. One vaccinated adult described how she had a history of getting vaccines and in this case, she had to ‘roll with the punches’ to be safe. She stated, ‘*I felt that if the Lord prompted me to do it, that was a good thing. … Yeah. I was ready. I was waiting until they got to my age bracket*.’ There were others who accepted the vaccine with doubt or lingering concerns. For example, one participant contracted COVID-19 and was eventually hospitalized. Because she had children she wanted to raise, she received the vaccine although she had concerns. She stated, ‘*It [the vaccine] does help protect against the virus. Still unsure and very uncertain with all the facts and all the minor details when it comes to the vaccine itself*.’ Others delayed the vaccine as they were seeking more information to make an informed decision. For example, one participant was in support of the vaccine but had so many questions on the vaccine that she decided to delay and observe others’ experiences. She stated, ‘*Oh, it’s [the COVID-19 vaccine] so new and it’s so scary and let’s just wait around and see how everybody else is*.’ A minute number refused the vaccine, indicating that they did not receive vaccines in general. One participant did not receive vaccines as an adult, wished he had not received vaccines as a child, and decided not to receive the COVID-19 vaccination. He stated, ‘*I don’t do vaccines for starters… I haven’t, I don’t do that…I feel I don’t need it [COVID-19 vaccination]*.’

#### 3.2.2. Varied Decision-Making Processes for COVID-19 Vaccination

**Figure 1:**
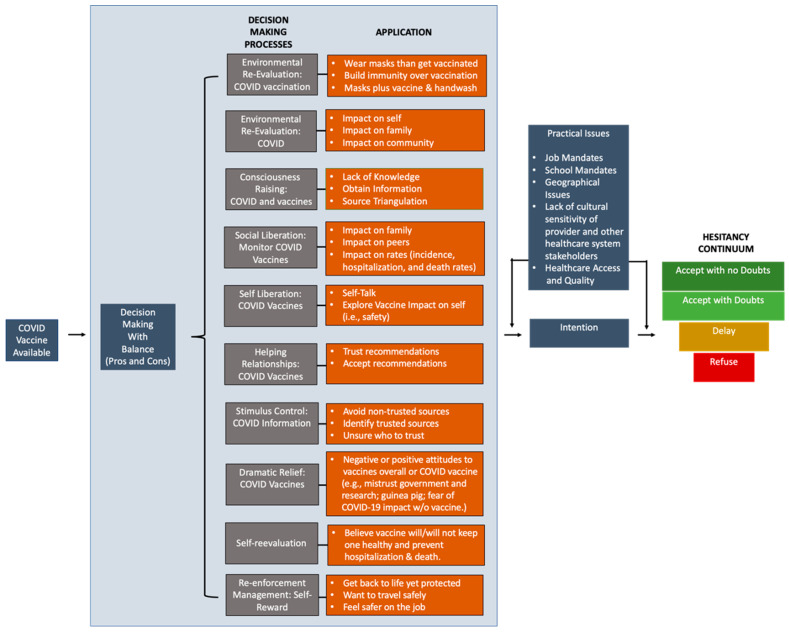
Decision-making processes model (DePC) model for COVID-19 vaccination. Based on Black Americans decision-making processes to be or not be vaccinated.

The decision to vaccinate or not was complex and required the consideration of multiple factors in almost all cases. Participants described different processes used in their decision-making (see Figure 1 for the decision-making processes for the COVID-19 vaccination (DePC) model among vaccinated and unvaccinated Black American participants). The application of decisional balance (i.e., weighing up the pros and cons) during their decision-making processes was the most mentioned process by participants. The common factors considered during the decision-making process were (1) knowledge and beliefs towards vaccines in general and COVID-19 vaccines specifically, (2) COVID-19 experiences of oneself and others, (3) social determinants of health, (4) experiences of others who received the vaccine, and (5) research and healthcare experiences. We apply TTM, HBM, and 3Cs to describe these processes and their application among these Black American participants. See Figure 1 for a summary.

Among participants who were vaccinated, many did an environmental reevaluation of how getting COVID-19 and the COVID-19 vaccination would affect them, their family, the community, and patients. For example, one vaccinated participant engaged in environmental reevaluation as it relates to getting COVID-19 and the vaccination. She stated, ‘*And so, for me, the benefits of being vaccinated far outweigh any of the risks, whether that be pain, fever, chills, whatever response the immune system can mount*.’ Some participants also evaluated whether one should wear masks and/or build immunity over vaccination. One participant did wonder whether engagement in multiple risk-mitigating behaviors (e.g., masks, vaccine, and handwash) reduced the likelihood of transmission compared to that of engagement in one or no preventive behaviors at all.

A few described social liberation in monitoring the effect of vaccination on family and peers, which increased their confidence in the vaccine. Others openly had discussions with family members, especially those in the medical field, friends, their providers, co-workers, and researchers. One participant perceived obtaining more information about their health and medications from a physician as an important step in evaluating the risks and benefits of being vaccinated. ‘*So, talk to your doctor about if this will interact with any of your medication, which [it] doesn’t by the way. But you know, if you’re worried that like, oh my thyroid medicine, now my thyroid is going to be off after I take this vaccine. That’s an excellent question. Make sure you write it down and bring it to your next doctor’s visit so that you can talk about it before you get your next shot*,’ the participant stated. A few even monitored the effect of vaccination on incidence, hospitalization, and death rates to determine its efficacy to inform decision-making.

All participants engaged in consciousness raising on COVID-19 and vaccines by accessing information sources. (See Section 3.2.5. to see more details on this process). To avoid misinformation or disinformation on COVID-19 and the vaccination, most applied stimulus control. Information sources that were identified were already deemed reputable, or participants compared different online sources to establish ‘*Google efficacy*’ among the sources. Many participants avoided non-trusted sources. A few participants did describe being unsure of who to trust and needing more guidance.

One participant even described how she engaged in self-liberation by telling herself to have the vaccine. Particularly, she explored the potential side effects of the vaccine on her body and then began to self-talk about the importance of the vaccine. Others engaged in self-reevaluation of whether the vaccine would or would not keep one healthy and prevent hospitalization and death. A few experienced dramatic relief as it relates to COVID-19 vaccines. Particularly, they discussed their attitudes about COVID-19 and the vaccination and the influence of their mistrust in the government, perception of being treated like a guinea pig, and fear of COVID-19’s effect on them without the vaccine. Yet, they understood the science and its importance. So, they chose to trust the process. Last, a few identified helping relationships such as those with a provider or researcher having an existing relationship with them or a great reputation within the Black American community. This relationship allowed the participants to trust and accept their recommendation.

Almost all participants described evaluating getting back to life, traveling, and working in the context of being vaccinated or not vaccinated. Being protected and safe were top priorities. Therefore, vaccination was viewed as a form of re-enforcement management: self-reward. A few participants indicated that they were unable to engage in a decision-making process as vaccination was required by their job. The pressure and force to get a vaccine diminishes individual freedom, as shared by this participant: ‘*But when your job forces you to get a vaccine, how many people are going to feel pressure to get a vaccine to pay their mortgage, to keep their lights on, to keep their kids in tennis shoes? All these things play a political role in what people will decide to do. Not their personal decisions, and it’s wrong. That’s robbing you of your “American freedom.” There’s no freedom if you have the choice of do we eat or do we do what we believe?*’

Among participants who were unvaccinated, most intended to get vaccinated. However, they were monitoring the vaccine’s effects on other individuals. A few stated that they were not going to have the vaccine as they did not believe in them and had not received them since childhood. One highlighted that if their child could not be vaccinated because the vaccine was not available for their age range, then one should not be vaccinated themselves. Similar to vaccinated participants, they engaged in consciousness raising by listening to conversations (e.g., researchers on the internet) or searched the internet and determined whether the source seemed trustworthy or like a ‘*shade tree conversation*.’ Validating COVID-19 information came from a review of multiple sources. A few participants even described how they engaged in self-liberation by telling themselves to have the vaccine, which was informed by the COVID-19 updates. However, it was not enough for them to get vaccinated.

#### 3.2.3. Motivators among the Vaccinated Individuals

Vaccinated participants in our study were motivated to seek beneficial outcomes while avoiding harmful ones. Almost all individuals had concerns about the COVID-19 vaccine prior to vaccine receipt. For example, a few participants discussed not fully understanding certain aspects of the vaccine such as ‘*emergency use authorization.’* Most thought the vaccine was rushed, leading to inquiries on its safety. Many stated that the vaccine was too new and wanted to ‘*wait and see*’ its short- and long-term effects on others. One participant was concerned that the data on vaccine efficacy and side effects were few for minoritized groups. Another participant mentioned a lack of knowledge on which information sources could be trusted. She stated, ‘*Because it’s still so new and it’s a still-evolving virus, I can’t say whether or not I’m confident that the actual vaccines are protecting us*.’ However, the motivators detailed below led study participants to the decision to get vaccinated.

**Perceived Benefits.** Participants understood that vaccines were designed to, and potentially did, protect the health of the vaccinated individual. For the COVID-19 vaccine specifically, they discussed the need to prevent or reduce the spread of the virus to loved ones and the community at large. For some, there were benefits to the vaccine, even though the vaccine is not always 100% effective. One participant stated, ‘*They’re still less likely for it to be as impactful or necessarily harmful. So, for example, my son’s mother was double vaccinated, but she still ended up getting it. But apparently it was no big deal. So, you may want to be able to highlight that as well. Yeah. Technically you could still get it, but the severity of it compared to somebody who’s not vaccinated is worth noting.*’ Another participant saw the benefits of the vaccine and the additive protection of taking precautions. ‘*If you wear a mask, if you wash your hands and do all that stuff, probably. Will you play a little bit better; will your game be a little stronger if you’re warmed up in your stretch and you practice? Probably. The COVID vaccine is going to give you that practice, give you the extra stretch, give you the boost, even though your body may be able to do it on its own right now*,’ the participant stated.

**Health Status.** Many participants discussed how their current health status influenced their decision. Participants and their peers with chronic conditions such as diabetes, hypertension, heart disease, and asthma were viewed as having a higher need for the vaccine to stay protected. There were similar perceptions among people with compromised immune systems. Some also considered risk factors such as lifestyle and social determinants of health when deciding whether to have the vaccine. Furthermore, these participants saw the risk of being unvaccinated as unacceptable, especially if one had poor health. ‘*Yes. A lot of our folks have diabetes and high blood pressure so that’s a question we get a lot, but I feel like they’re much easier to convince because they know that they’re more likely to die from COVID, so even if the vaccine has side effects… Because of that early messaging in 2020 about people with autoimmune diseases and diabetes and high blood pressure should really be careful from COVID is now… I feel like they’ve been kind of the most receptive group to the vaccine*,’ one participant stated.

**Trust in Science and/or Vaccines.** Others highlighted their trust in science and researchers was based on their history of being effective in disease prevention and reducing severity. For example, a participant recalled when the polio and smallpox outbreak occurred, creating fear and perceived lack of control. Yet, the vaccines were effective. Therefore, they were not ‘*anti-vaxxers*’ and had history of receiving vaccines. One participant stated, ‘*I don’t want to say it’s the more the merrier, but I’m just prepared in any way, shape, or form to get another booster as needed. So, I did put my trust in science. … I was really excited about the booster coming out because I felt like the vaccine itself was effective*.’

**Individual/Interpersonal/Communal Experiences**. Getting vaccinated was motivated by how others felt about vaccination and how one thought vaccination might affect other people. Particularly, getting vaccinated was a way to protect oneself as well as family members and the community at large from the virus, a form of altruism. One participant stated, ‘*I don’t want to contract this from a patient or from my healthcare setting and then pass it to my parents. So that just seemed to me like if there’s anything, they made me, so if there’s anything that I could do to protect them, even at the expense of my own health, I would do it*.’ Other participants mentioned family, coworkers, and friends as people influencing their decision to get vaccinated. Many individuals had a personal experience including contracting the virus, or a friend or family member had contracted and, in some cases, died from COVID-19. These experiences allowed them to understand the severity of the disease and invoked fear of death. Also, the positive experiences of family and friends when they were vaccinated (i.e., little to no side effects) were motivating. Being able to see that the vaccine did not cause ‘*severe*’ side effects affected decision-making.

**Need To Create a Sense of Normalcy in Life.** Travel and work were important factors in deciding to be vaccinated for a handful of participants. Particularly, participants desired to ‘*get-back-to-life*’ or feel safe as day-to-day activities were resumed. A participant stated, ‘*Well, I was planning on traveling. That was kind of the real big push. I was already planning to take it, but it was just going to be one of those things. And I got vaccinated in April. Yeah, so that would be the main reason. Maybe the secondary one is health and safety because I’m around a lot of people, and because of my work and exposure*.’ The provision and/or mandating of the vaccine at work led to the decision to have the vaccine.

#### 3.2.4. Barriers among Unvaccinated Individuals

Almost all participants who were unvaccinated for COVID-19 had positive views of vaccines, particularly their ability to build one’s immunity to prevent or reduce the severity of disease. Only a few participants had not had vaccinations since childhood. Participants who were unvaccinated had several issues with the COVID-19 vaccine, as described below.

**Vaccine- and Vaccination-Specific Issues.** The newness of the vaccine along with not knowing its safety profile was cited across all participants as a concern. These concerns were driven by the speed of the vaccine’s development. Some participants also highlighted perceived ineffectiveness of the vaccine against COVID-19 infection, hospitalization, and even death. ‘*So, what’s the essence of you getting vaccinated against the virus that you can eventually contract again?*,’ one participant stated. Many participants also questioned how vaccines work, the rationale for the short period of vaccine testing prior to its availability to the public, and its ingredients. A concern highlighted by most participants was the ‘*multiple doses*’ required. The idea of awaiting FDA approval while allowing individuals to have the vaccines under emergency use authorization (EUA) baffled some participants. Particularly, most participants did not understand why so many boosters were given and how fast they were being recommended for use. This raised a ‘*red flag*,’ making some feel that ‘*it’s bigger than COVID-19*.’ One participant emphasized, *‘Now that they’ve come out with this booster and they’re saying that people are not fully vaccinated now until they get the booster and I’m like, “Okay, how many boosters are you going to say we need to get?” That’s another red flag. I mean, all of our previous vaccines basically have been a one and done or a 10-year booster, not a booster 6 months after, we just gave the EUA now you got to get another booster. I don’t know about all that. We just don’t have a lengthy enough review of your review period to know how this is really going to affect our bodies*.’

Participants’ confidence in the vaccine was also reduced by the perceived short-term effects (e.g., infertility and sterility, illness, death) and the unknown long-term effects post vaccination. This was commonly mentioned among individuals with underlying medical conditions. ‘*Yeah, that was a huge concern for me, being a 33-year-old who has never had a child. My thing was I don’t want to go in and get this shot and then find out I can’t have kids. But after more research, I did find out it wasn’t affecting fertility rates for women and the number of women who still got pregnant after getting vaccines and things like that*,’ one participant stated.

**Lack of Information, Misinformation, and Disinformation.** Low levels of confidence in the vaccine were further fueled by individuals’ lack of knowledge, misinformation, and disinformation on COVID-19 and the vaccine. The infodemic surrounding COVID-19 and the vaccine made it difficult for these participants to identify trusted information. This was primarily due to social media and the internet. However, sometimes family and/or friends provided information that was suspect. Receipt of the recommendation to not receive the vaccine or poor communication surrounding the vaccine negatively influenced some participants’ decision-making.

**Mistrust and Distrust in COVID-19 Key Players.** There was existing mistrust and distrust in the healthcare system and providers, researchers and the research process, the government, and/or pharmaceutical companies. The long-standing history of discrimination in healthcare (e.g., lack of access to healthcare, minimal treatment) and lack of culturally sensitive providers were highlighted by a few participants. The historical abuse of Black Americans in research and the fear of being treated as guinea pigs were also commonly mentioned by these participants. Many thought pharmaceutical companies were motivated by profit and that the drugs produced were ‘pushed’ by providers. Last, the persistent perception of racism exhibited through government decisions, particularly at the national level fueled mistrust and distrust during the COVID-19 pandemic. Because of these existing barriers, their involvement in vaccine development led many to be hesitant or refuse to have the vaccine. One participant stated, ‘*Well, for starters, it’s just the mistrust of the medical field and African Americans, to me in general, it’s really my apprehension behind it*.’ A few felt that the vaccine had been politicized.

**Attitudes Towards COVID-19 and the Vaccination.** Many concluded that there was not sufficient risk of poor outcomes to justify being vaccinated. Others argued that the vaccine is not necessary because they already had COVID-19, or COVID-19 was a mild disease. One participant further perceived COVID-19 death rates as being low compared to those of other diseases. Another did not fear COVID-19. She stated, ‘*It wasn’t severe. I didn’t even lose taste or smell. I just got, like, a runny nose for a couple days*.’ A few participants felt *‘safer’* with COVID-19 than getting the vaccine, or that natural immunity was best. Some participants also believed that their immune system was so strong that they did not need the vaccine. This was due to their history of rarely getting sick or to their taking steps to strengthen their immune system. ‘*One thing that I’ve learned with COVID is to build your immune system and that’s what I have been doing, is building my immune system. I’ve been taking multi gummies for two years and so I started taking it even before COVID hit*,’ the participant stated. Taking precautions like physical distancing, handwashing, and masking was another source of complacency for some participants, even those with underlying medical conditions.

**Access to Testing and Vaccine Information Sites.** The location of COVID-19 testing and vaccination sites made them convenient or inconvenient, depending upon the distance from home and work. Competing priorities were also found to make vaccination more inconvenient. One participant highlighted, ‘*It was just kind of like a time conflict between their [children’s] school, my schedule, just getting them back in there [doctor’s office] or to a pharmacy to get them vaccinated. Although I’m still unsure, I’m trying to make preparations to get that, work towards that*.’ One participant further highlighted that some populations, including older adults, may be harder to reach with information or to administer the vaccine because of their experience of being placed on the waiting list and never receiving information on next steps for vaccine administration.

#### 3.2.5. Retrieving and Navigating Vaccine Information with the COVID-19 Infodemic

All participants desired and sought information related to the COVID-19 vaccine, regardless of their vaccine status. Information was critical to the decision-making processes among participants as they were seeking answers to their questions and making sense of their communication environment. Participants had to navigate the information from various sources and channels to inform their decision-making, and to distinguish whether they perceive information to be true or false. Trust provided a foundation for the assessment of communication sources, content, and channels. Below, we further expound the intersection of navigating sources, accessing information, and identifying trusted messengers. We also highlight COVID-19 vaccine-related communication needs for Black American participants. 

**Navigating Answers to Outstanding Questions.** Participants sought information and education from their environments to fulfill their informational need on COVID-19 vaccines. When seeking answers, some participants stated that they wanted access to as much information as possible for informed decision-making. One participant stated, ‘*I need full disclosure. I don’t need no misinformation, half information, information held back, whatever. I need to know everything if you want me to put this into my body. Because if it’s something that can do me more harm than good in the long run, I need to know that*.’ Participants also noted questions for which they wanted answers. They further alluded to areas of confusion. ‘*And so, what is an mRNA? A lot of people are not going to know what that means. And when they see that, the first thing that they always say or what I’ve heard people say is, “Oh, that’s when y’all use those aborted babies or y’all are using dead babies to make this vaccine. And that’s the reason why I don’t want it.” And there again, there’s no explanation. There’s no education of what an mRNA vaccine is*,’ one participant described.

Multiple information sources were used to inform their assessment of the COVID-19 vaccine. Information sources included discussions with family and friends, watching local and national news, conducting internet searches, and engaging with social media posts. Some participants expressed their confidence in determining the reliability of these sources. They stated they discarded or ignored information they perceived to be half-truths, false, or conspiracy theories. One participant highlighted, ‘*I’m not going to subscribe to any of those crazy-ass conspiracy theories: this makes you magnetized and sterilizing you and stuff like that. To me, that’s retarded. I mean, I just don’t think that makes a lot of sense. I think that it makes a lot of sense for the government to want to do that or pharma companies to do that. Like if they’re killing the population, they’re killing their money, they’re killing the taxable people. It doesn’t make sense*.’

**Accessing the Internet for COVID-19 Vaccine Information.** The internet is readily accessible to nearly all population segments. It provides continual access to multiple information sources through various channels. Social media platforms (e.g., Facebook and Twitter) were commonly accessed for COVID-19 vaccine information. They were used by participants to view individuals’ personal experiences with COVID-19 and the vaccination. Social media was a tool cited as being used by most participants, and they perceived it to be used by others. Participants discussed learning about the vaccine and related research studies on various social media platforms. One participant stated, ‘*Cause I have seen a couple studies on TikTok. So, I always think of that as my number one. And then Instagram, Facebook, the traditional ways, or LinkedIn. A lot of people do LinkedIn these days, ‘cause everyone’s looking for a job*.’ While social media was commonly used, it was not above criticism. One participant believed individuals should not be swayed by the information on social media and the news, but by one’s own research. Internet search engines such as Google were used by some participants to gather COVID-19 vaccine information. It was also used to confirm or refute the information found on other social media platforms, like Facebook. Here, a participant describes that process:

*‘Sometimes I just see them on Facebook, like, this whole awareness stuff. So, I see stuff on Facebook, and I have searched of the effects, the side effects of the vaccine. I do know the short-term side effects, but the long-term side effects, and mutations, and stuff like that. But it was all basically positive stuff. There’s no long-term side effects and stuff like that. So, I found details on Facebook. I found details on Google when I searched.’*

**Trusted Messengers.** Identifying and using information sources and their related messages was done by filtering through their information environments. Use of the information was also based on the degree of trust these participants had in the source of the information. Characteristics of a trusted information source were to have a background in the topic (e.g., a researcher or provider), state statistics, and/or data, and provide ongoing updates. One participant stated, ‘*I always say trust but verify. Sure, you could trust what people say, but verify it. Where is that stuff coming from? Is it just made up? Is it just something that you’re hoping for or is it actually real? And then if it’s real, we can have a conversation about it*.’ Governmental websites (e.g., Centers for Disease Control and Prevention, Food and Drug Administration, and PubMed) were commonly accessed to obtain COVID-19 rates (i.e., transmission, prevalence, and mortality rates) and the safety profile of the vaccine (e.g., side effects). Yet nearly all participants expressed the least trust in the government, especially government officials. Participants did not want the government involved in their healthcare decision-making based on their overall priorities and policies. This distrust was further fueled by the historical, healthcare experiences of Black Americans.

The level of government (i.e., local, state, and federal) was considered when participants reported their trust. Many stated that they were more trusting of local- and state-level governments because elected officials were more accessible to their constituents. This proximity seemingly provided some exception to their overall lack of trust in the government. ‘*I think that’s kind of closer to home, so I feel like those, they can actually relate more to the community, but still, it’s politics. So, my trust level is still very low, even though I feel like they’re more accessible*,’ stated a participant. Healthcare providers, such as physicians and nurse practitioners, were deemed trustworthy by some participants, even among the unvaccinated. Participants had a relatively positive view of their respective healthcare providers and trusted their assessments related to their health. One vaccinated participant discussed how racial concordance with their provider fostered trust and acceptance of healthcare recommendations, including COVID-19 vaccination. A participant highlighted, ‘*My doctor is Black. And so, I have more trust in the doctors that look like me just because I feel like there’s less of a bias and more vigor or I guess more diligence and due diligence in actually coming up with solutions for any ailments I may come up with…*’ Some participants, while they trusted their providers, stated they did not solely use information from their providers to make healthcare decisions.

#### 3.2.6. Parent Perspectives on Vaccinating Children

All parent participants (*n* = 14; *n* = 7 vaccinated and *n* = 7 unvaccinated) thought they were acting in the best interest of their children. The approach to COVID-19 vaccine uptake for their children was guided by their own vaccine decision. Regardless of vaccination status, parents’ concerns about the benefits, safety, and efficacy of the vaccine were amplified as they considered their duty to protect their children and their overall health. ‘*As a parent, I ultimately made that decision that I would love to be here to raise my children, so it was very important that I did everything that I could within my power to remain safe and to protect me with aiding myself. Although I know it’s not a cure, but it does help protect against the virus. … it was just basically I felt it was an obligation, as this privilege to be a parent, that I do what I can to protect myself, and of course my family, against the virus*,’ one vaccinated parent stated.

Some vaccinated parents named their children as motivators to get themselves vaccinated. They wanted to prevent possible transmission to their child(ren). Others had a bad personal experience with COVID-19, motivating them to want to live to raise their child(ren). For unvaccinated parents, there were expressions of uncertainty or a lack of clarity on COVID-19 vaccine information and communication for informed decision-making. ‘*I just feel like there’s not enough clear-cut answers to what it does. You hear all the propaganda on commercials and social media, and then your doctor chimes in their personal opinion. And nobody can give you definite answers. It’s too many unknowns*,’ one participant stated. Others expressed concern about their perception of the lack of information. A participant emphasized, ‘*I don’t think there’s enough information out about the side effects, and it seems to me it’s going to be like the flu shot. I’ve never received the flu shot nor have my children*.’

**Apprehension.** Both vaccinated and unvaccinated parents expressed apprehension about the vaccine for their children. Their greatest concern was the vaccine’s potential for short- and long-term side effects. To actively address this, one vaccinated parent shared how she discussed the COVID-19 vaccine with her child using information from her provider and their child’s pediatrician. ‘*My older children, we’ve had the discussion about them getting vaccinated to protect them against possibly contracting the virus, it’ll help them to be able to fight it off. As much information as I try to provide or speak with them, although I’ve obtained that from my physician, as well as the pediatrician, they’re just not comfortable getting the vaccine, although they get the flu shot every year. It’s just a lot of apprehension*,’ stated the participant. Among some unvaccinated parents, their apprehension about the vaccine and their children was relayed anecdotally.

**Role of the Provider.** All parents perceived healthcare providers to play a fundamental role in vaccine uptake through administration and addressing hesitancy. They felt that they should act as vaccine educators and advocates. Most parents trusted their children’s providers because of their long-term relationships. One vaccinated parent stated she trusted her child’s pediatrician recommendation and the information provided on the vaccines’ effectiveness for herself and her child. However, she recounted an experience where she had to advocate for her child to receive a vaccine when the provider refused. An unvaccinated parent stated that her child’s provider did not mention or suggest the COVID-19 vaccine. She stated, ‘*Then the doctors, they don’t try to persuade you. They would just mention it, but my first child, when she went, they didn’t even mention [the COVID-19 vaccination], “Are you going to get it for her or not?” So, it was almost like… because would we go in for physicals and stuff, they tell you about the ‘flu vaccine a lot, or they tell you, “Oh, it’s time for her…” It’s almost… it’s like some stuff is mandatory and this is not mandatory. So that’s like, “Well, they’re not pushing it. Why should I worry about it?” but, yeah, all the other vaccines, it’s a mandatory thing [for school]*.’ Because she had a long-standing relationship and trusted her child’s provider, this was a missed opportunity for her child to have the vaccine.

## 4. Discussion

Vaccine hesitancy exists along a continuum [31]; our study confirms this phenomenon among the Black Americans, including parents, in our study. Our study demonstrates the complexity in the decision-making process among COVID-19 vaccinated and unvaccinated participants, although decisional balance was used by both groups. This finding was similar among parent participants. Understanding the processes of behavior change is critical in encouraging a change in behavior, in this case, increasing COVID-19 vaccine uptake [32]. One important finding is that some participants used similar processes that led to inverse decision-making. For example, some participants noted how consciousness raising via vaccine receipt of family and peers prompted them to get vaccinated; yet a parent did not have the vaccine when offered because a vaccine was not available for her child. Understanding the decision-making processes for vaccine uptake is critical to better align the intervention strategies aimed at moving undecided adults and parents toward acceptance of the vaccine [33].

To our knowledge, this is one of few studies to explore the factors influencing COVID-19 vaccine receipt among Black Americans. The desires to ‘resume life’ and to protect oneself and others were the most cited reasons for uptake among adults and parents. Similarly, Galanis [34], Jang [35], and Stoner [36] found that COVID-19 vaccination was positively associated with viewing the vaccine as safe, effective, and important to protect oneself and others and return to normal life among adults and parents. Interestingly, almost all participants highlighted their trust in science and the history of the vaccines, which filtered into their positive views towards the vaccines. In a study by the Pew Research Center, approximately eight out of ten Black Americans had a ‘fair amount of confidence,’ with at least some trust in medical researchers [37]. These findings warrant further exploration to reveal unidentified strategies to promote confidence and build trust in medical researchers and the process. Last, the COVID-19 and vaccination experiences of others, particularly those of family and friends, were critical in the decision-making of vaccine acceptors. Such individuals have been found to play a role in healthcare decision-making for many years. Therefore, strategies should include the engagement of these individuals to promote COVID-19 vaccine uptake.

In relation to factors influencing decision-making among vaccine non-acceptors, three main categories emerged, reflecting the 3 Cs model of vaccine hesitancy. Similar to existing research [10,18,38], we found that low confidence was primarily due to a lack of understanding of the vaccines’ safety and efficacy, and concerns of the vaccines’ side effects and being new. It was not surprising that mistrust and distrust in research and the process emerged as factors lowering confidence of many participants, including parents. Similar to Bateman et al. [39], we found that the long-standing history of research abuses among Black Americans continues to impact trust in the process and uptake of preventive health behaviors like COVID-19 vaccine. Strategies that could potentially increase trust include the acknowledgement of systemic racism, increased access points for research, and use of trusted leaders and messengers to provide information [40]. It was common for those unvaccinated to demonstrate complacency in their decision due to the perception of low disease risk, especially if taking secondary precautions or having a strong immune system. Because the perceived low risk for COVID-19 continues to emerge as a reason for non-acceptance of the vaccine [10,38,41], education efforts are needed to demonstrate the importance of vaccination and the long-term consequences if not vaccinated. The lack of convenience was evident in the inequitable distribution and allocation of the vaccine throughout the pandemic, especially in early stages [42]. Our study confirmed these findings, with a few participants describing their enthusiasm being hampered by the barriers faced when trying to access the vaccine. Collectively, these findings demonstrate that although barriers exist in many areas, building confidence is critical for vaccine acceptance.

Risk communication is critical for pandemic preparedness, including educating on the opportunities to prevent, quickly respond to, and recover from a pandemic [43]. Little is known about the needs of Black Americans as it relates to risk communication, including the area of COVID-19 vaccination. For example, Cunningham-Erves [17] described the use of community engagement to develop a theory-based, culturally appropriate message library to promote vaccination. As the first study to explore communication needs by vaccination status, participants had similar messaging needs and used the same types of information sources, which were trusted and/or validated through a review of several sources. For example, governmental websites were used to learn of the vaccine’s safety profile, and social media was used to learn of individuals’ COVID-19 or vaccination experiences. Communication needs lingered post vaccination, suggesting that education does not end with vaccination. Furthermore, the use of multiple stakeholders to inform the study and its implementation was critical in understanding the communication needs of this high-risk group. Collectively, our strategy and findings support the need for trusted messaging by multiple stakeholders before and after COVID-19 vaccination. This is critical as current and future risk communication plans are in development phases.

This study is not without limitations. Similar to other multi-method studies, the qualitative findings are not generalizable to other racial and ethnic groups, or to other geographical areas. However, the study is exploratory and can be used to inform the development of future studies, including a quantitative study to extend this work and health education and promotion initiatives. The study population was mostly composed of Black American females with a college degree or higher. There is a potential for researcher bias to affect data collection and interpretation. However, researcher triangulation and intercoder reliability were applied.

## 5. Conclusions

Vaccine hesitancy, especially among Black Americans, presents major challenges to COVID-19 vaccine uptake and to reducing the related disparities in hospitalizations and deaths. In this study, we identified the decision-making process, motivators and barriers, and communication needs and strategies among vaccinated and unvaccinated Black Americans. Ultimately, these findings can aid in the development of culturally appropriate interventions among multi-level allies and partners to improve vaccine uptake. Future research should further explore how factors influencing decision-making can lead to divergent outcomes for COVID-19 vaccination. Further exploration is also needed into the emergent themes to understand how intervention strategies align to build on motivating factors and overcome barriers.

## Figures and Tables

**Table 1:** Socio-demographic characteristics of participants by vaccination status.

	Vaccinated *n* = 16 (%)	Unvaccinated *n* = 14 (%)
Sex, *n* (%)		
Male	4 (25)	2 (14)
Female	12 (75)	12 (86)
Age in y (mean, SD)	38.9 (10)	38.3 (9)
Education level, *n* (%)		
Some college or lower	4 (25)	4 (29)
College or higher	12 (75)	10 (71)
Household income, *n* (%)		
$40,000 or less	4 (27)	5 (39)
$40,001 to $80,000	4 (27)	2 (15)
Over $80,000	6 (40)	4 (31)
Prefer not to answer	1 (7)	2 (15)

Note: The household income item for the vaccinated column does not add up to 16 as one participant did not answer the question.

**Table 2:** Participant’s hesitant attitudes toward COVID-19 vaccines.

	Vaccinated *n* = 16 (53%)		Unvaccinated *n* = 14 (47%)		*p* Value
	Disagree	Agree	Disagree	Agree	
I wonder if getting many different vaccines could cause harm to my health.	10 (77)	3 (23)	3 (23)	10 (77)	0.017 *
I wonder if natural immunity is better than getting the COVID-19 vaccine.	10 (71)	4 (29)	2 (15)	11 (85)	0.001 *
I wonder if the vaccine could cause serious health problems.	4 (31)	9 (69)	3 (23)	10 (77)	1.000
I wonder if the COVID-19 vaccine is effective in preventing the COVID-19 virus.	11 (69)	5 (31)	4 (33)	8 (67)	0.125
I wonder if the COVID-19 vaccine is needed to prevent the COVID-19 virus.	6 (38)	9 (62)	5 (39)	8 (61)	0.934
I wonder if I should wait to have the vaccine to see how it works.	7 (44)	9 (56)	4 (33)	8 (67)	0.705
I wonder how fast the COVID-19 vaccines were made.	5 (31)	11 (69)	3 (23)	10 (77)	0.697
I have concerns that the government was too involved in the development of COVID-19 vaccines.	11 (69)	5 (31)	5 (38)	8 (62)	0.103
I wonder if the COVID-19 vaccine is too new.	7 (44)	9 (56)	1 (8)	12 (92)	0.044 *
I wonder if the COVID-19 vaccine is safe.	7 (44)	9 (56)	2 (15)	11 (85)	0.130

Note: These categories were collapsed from the four-point Likert scale based on agreement. Some questions may not add up to 14 due to missing data. * *p* < 0.05.

**Table 3:** Self-reported motivations for COVID-19 vaccination among the vaccinated participants (*n* = 16).

Motivators for Vaccination	*f* (%)
Reading/listening to a news story discussing COVID-19 vaccine trials.	7 (44)
A friend or family member being diagnosed with COVID-19.	6 (38)
A friend or family member passed away due to COVID-19.	5 (31)
Having a conversation with friends or family about whether to get a vaccine.	5 (31)
A friend or family member receiving the COVID-19 vaccine.	5 (31)
Reading or hearing a news story about results of those already vaccinated.	4 (25)
Wanting to visit family or friends but not being able to without a vaccine.	4 (25)
Wanting to return a work or school but not being able to without a vaccine.	4 (25)
Wanting to travel but not being able to without a vaccine.	3 (19)
Being directly contacted by a health professional with information on how to get vaccinated.	2 (13)
Watching a commercial or a PSA about how to get vaccinated for COVID-19.	0 (0)
Seeing a public figure get a vaccine.	0 (0.0)

**Table 4:** Self-reported barriers to COVID-19 vaccination among the unvaccinated participants (*n* = 14).

Barriers to COVID-19 Vaccination	*f* (%)
Heart problems	8 (73)
Infertility	7 (64)
Speed of development	5 (46)
Blood clots	5 (46)
Autism	3 (27)
Gives me COVID-19 virus	3 (27)
Microchip within the COVID-19 vaccine	2 (18)
Change of my DNA	2 (18)
Warp speed	1 (9)
Guillain-Barré Syndrome	1 (9)

## Data Availability

Due to the confidentiality agreements, supporting data cannot be made openly available.
